# Management of TB/HIV co-infection: the state of the evidence

**DOI:** 10.4314/gmj.v54i3.10

**Published:** 2020-09

**Authors:** Kwasi Torpey, Adwoa Agyei-Nkansah, Lily Ogyiri, Audrey Forson, Margaret Lartey, William Ampofo, Joseph Akamah, Peter Puplampu

**Affiliations:** 1 Department of Population, Family and Reproductive Health, University of Ghana School of Public Health; 2 Department of Medicine and Therapeutics, University of Ghana Medical School; 3 Department of Virology, University of Ghana Noguchi Memorial Institute of Medical Research

**Keywords:** Tuberculosis, management, HIV, MDR TB, Ghana

## Abstract

Tuberculosis (TB) and HIV are strongly linked. There is a 19 times increased risk of developing active TB in people living with HIV than in HIV-negative people with Sub-Saharan Africa being the hardest hit region. According to the WHO, 1.3 million people died from TB, and an additional 300,000 TB-related deaths among people living with HIV. Although some progress has been made in reducing TB-related deaths among people living with HIV due to the evolution of diagnostics, treatment and antiretroviral HIV treatment, multi drug resistant TB is becoming a source of worry. Though significant progress has been made at the national level, understanding the state of the evidence and the challenges will better inform the national response of the opportunities for improved patient outcomes.

**Funding:**

None

## Introduction

Tuberculosis (TB) is among the leading causes of mortality worldwide (ahead of human immunodeficiency virus, (HIV) from infectious diseases. In 2017, tuberculosis resulted in 1.6 million deaths globally. Although its mortality fell by 37% from 2000 to 2016.^1^,^2^,^3^

Tuberculosis ranks ninth among the leading causes of diseases globally. The incidence of TB in 2017 was 10.0 million, with almost two-thirds occurring in just eight countries (China, Philippines, Pakistan, Nigeria, South Africa, Bangladesh, Indonesia and India).^2^ Fifty-four million deaths have been prevented between the year 2000 and 2017 because of improved diagnosis and effective management of TB worldwide. The WHO European and African regions have experienced the most rapid declines in mortality; 5% and 4% respectively from 2013 to 2017. The End TB Strategy is one of the time-specific strategies that set targets for prevention, care, and control of TB. The WHO declaration of TB as a global public health emergency in 1993 led to these high-level interventions. The world has experienced a gradual improvement of the situation ever since.^4^ In 2015, the WHO proclaimed to have accomplished the goals set in target six of the Millennium Development Goals (MDGs) which aimed to halt and reverse TB incidence.

Consequently, the goals of the Stop TB Partnership (which precedes the End TB Strategy) coined out of the MDG target six were also met.^5^ The milestones of the End TB Strategy are to reduce the absolute number of TB deaths by 35% and 75% by 2020 and 2025 respectively and to achieve a decline in TB incidence rate by 20% and 50% respectively by 2020 and 2025.^6^ Currently, the global mortality rate (among HIV-negative people) declines by 3% yearly whiles the incidence rate reduces by 2% yearly.^2^ In order to meet the 2020 milestones, the decline in the incidence rate and mortality should be 4 – 5 % and 10% every year respectively.^2^ Globally, sub-Saharan Africa (SSA) region is an area with high TB/HIV associated deaths and morbidity. SSA has 12% of the world's population but accounted for 30% of the 9 million TB cases and over 250 thousand TB-related deaths. Coupled with this, is the high prevalence of HIV in this region leading to 50% of these patients being co-infected with HIV.^7^

In 2016, the WHO African region contributed to 25% of the total number of incident cases of both TB mono infection and TB/HIV dual infection globally. Nigeria reported 407 cases per 100,000 population in that same year contributing to 8% of the global incidence compared to 322 cases per 100,000 in the previous year.^8^

This may, however, be an underestimation because only 15% of TB cases were notified in 2015.^8^ Among HIV infected persons, TB is the leading cause of death, especially in resource-limited settings like Africa. Several factors have been attributed to the high prevalence of TB in Africa including HIV, some environmental and social factors like poverty and poor nutrition.^9^

Contributing to the burden of this disease is the emergence of Multi-Drug Resistant TB (MDR-TB),^10^,^11^ caused by *Mycobacterium tuberculosis* (*M.tb*) strains resistant to at least rifampicin and isoniazid; and of Extensively Drug-Resistant TB (XDR-TB). XDR-TB organisms are resistant to rifampicin, isoniazid, plus any fluoroquinolone and at least one of the three injectable second-line TB drugs (amikacin, kanamycin, capreomycin). Weaknesses in health systems, lack of resources and ineffective treatment regimens coupled with other operational treatment challenges all contribute to the low cure rates.

The national TB prevalence survey concluded in 2014, reported a TB incidence 165/100,000 population in the country. The latest report shows a decline in the incidence of TB in Ghana (156/100,000 population) since 2014.^7^ On the contrary, mortality among HIV-negative TB patients remains the same (36/100,000 populations from 2014 to 2016) and that among HIV-positive TB patients has rather increased from 16 to 20 deaths per 100,000 population in the same period.^12^,^13^

National TB case notification rates have declined from about 62/100,000 population in 2008 to 54/100,000 population in 2016. A similar trend is occurring at the regional levels. The Greater Accra, Western, Eastern and Volta Regions recorded the highest case-notification rates in 2016, higher than the average figure for the whole country, whiles the Northern Region recorded the lowest rate 24/100,000 population. The rate for Upper West is 48/100,000.^14^

The first pillar of the End TB Strategy, which is “Integrated, patient-centred care and prevention” has four key components. These are the early diagnosis of TB, including universal drug susceptibility testing, and systematic screening of contacts and high-risk groups; treatment of all people with TB, including drug resistant TB, and patient support; collaborative TB/HIV activities and management of comorbidities; and preventive treatment of persons at high risk and vaccination against TB. Reduction in TB incidence and mortality require widespread access to diagnosis and care and treatment to substantially lower the risk of developing TB in people who have a latent TB. Ghana is one of the countries that has implemented this in her policy.

HIV infection was discovered in the early 1980s.^15^ About 77.3 million people globally have had the infection with 35.4 million deaths since its discovery. Prevention strategies have helped to decrease the incidence of HIV infection by nearly half (47%) since 1996. Also, the uptake and scale-up of highly active antiretroviral drugs (HAART) have contributed to the decline in AIDS-related deaths by 51% since 2004. In 2017, 36.9 million people worldwide were estimated to be living with HIV.^16^

HIV infection spread rapidly since the first case was diagnosed in Ghana in 1986.^17^ The trend of new infections since year 2000 has decreased steadily from 30,000 to 20,000 in 2016. Death rates have also taken a similar path from about 30,000 to 15,000 between 2000 and 2016.^18^ About 310,000 adults and children are living with the infection in the country, of which 130,000 are estimated to be on antiretroviral therapy (ART). The estimated adult (15 to 49 years) prevalence is about 1.7%.^15^ HIV/AIDS has orphaned about 270,000 children 17 years or younger in the country. Among the regions, the Volta and Brong-Ahafo Regions recorded the highest prevalence of 2.7% whilst the Northern Region had the lowest (0.7%).^14^ The proportion of HIV subtype 1 in Ghana is 98.5%, and that of dual infections of HIV subtypes 1 and 2 is 1.5%.^19^

Ten percent of the 10.4 million TB patients worldwide also had HIV infection.^20^ About 74% of co-infected persons live in SSA. About 390,000 and 374,000 deaths occurred as a result of the co-infection globally in 2015 and 2016 respectively.^7^

HIV co-infection increases one's risk of developing active TB by about 19 times compared to HIV mono-infection. Co-infected patients are more susceptible to multidrug-resistant or extensively drug-resistant TB.^20^ Testing of all TB and HIV- positive patients for the dual infection and timely initiation of therapy are essential in reducing mortality and the burden of TB/HIV co-infection.^7^

The treatment coverage of tuberculosis in Ghana reached 100% in the year 2000.^21^ Much effort had been put into active and passive case finding of TB and subsequent linkage to care. The incidence of the dual infection in 2016 was 35/100,000 population; a reduction from 42/100,000 population in two years.^12^,^13^ Nearly a quarter of roughly 15,000 TB cases notified with known HIV status tested positive in the three years preceding 2017, with 1,200 (39 – 43%) of them receiving ART.^12^,^13^,^22^ The estimated proportion of TB/HIV patients in Ghana receiving ART is less than 25%.^3^ As pertains in other Sub-Saharan African countries, the effect of HIV on TB has led to challenges in the control of the infection. Ghana is classified as a country with a high burden.

Hospital studies have also shown that the prevalence of HIV in TB patients is approximately 25–30% and that as many as 50% of patients with chronic cough could be HIV positive.^23^

We present in this paper, the state of the art in TB/HIV dual infection management, taking into consideration the evolution of diagnostics, treatment, and their accompanying challenges. It highlights the situation in Ghana and opportunities for management of TB-HIV for improved patient outcomes.

## Methods

We searched for full-text articles published from 2000 to 2018 from three bibliographic databases, namely PubMed, ScienceDirect and Google Scholar. The keywords used as search terms were TB, HIV, diagnosis, treatment, Ghana, Africa. Published studies and treatment guidelines on the diagnosis, management and treatment of TB/HIV co-infection were selected for inclusion. Only peer-reviewed articles published in English were selected.

## Results

### Diagnostics

#### Microscopy

Sputum smear microscopy is one of the traditional diagnostic methods for detecting the presence of mycobacteria tuberculosis complex species (MTBC). The presence of the thick cell walls of MTBC is stained by acidic reagents used in the Ziehl-Neelsen method, which gives them the ‘acid-fast’ characteristic and can be identified under a light microscope. The method of staining was first used by Robert Koch and later led to the discovery of Mycobacteria tuberculosis in 1882.^24^ The tests can be performed at peripheral and high-level laboratories and are cheap to operate.^25^

The light-emitting diode (LED) fluorescent microscope is an innovation that simplifies viewing of fluorescent acid-fast bacilli (AFB) stains that were previously difficult to do so due to handling methods required.^26^ LED microscope replaced conventional microscopy (per 2010 WHO recommendations) as the preferred tool for carrying out sputum smear microscopy.^27^ The added advantage of using fluorescence is the increased specificity (10% higher) compared to light microscopy.^26^ Previously, three specimens were collected at different times of the day for examinations. However, studies showed that ‘spot’ collections of two specimens were enough to make a confident diagnosis since a third specimen does little to increase the specificity of the test.^28^

It is recommended that two consecutive sputum specimens of an individual must undergo smear microscopy to identify smear-positive patients correctly. Sputum smear microscopy is the core method for diagnosing smears positive pulmonary TB in Ghana. It is usually preceded by clinical screening and a chest x-ray. CD4 counts when available can help clinicians interpret presentation more accurately, as non-classic presentations may accompany lower CD4 counts.^23^ Over-reliance on sputum smear microscopy which is a low-sensitivity visual diagnostic test that cannot determine drug resistance has side-lined individuals whose illnesses are characterised by a lower TB bacillary load, such as children and individuals with HIV and diabetes, and also those with extrapulmonary or drug-resistant tuberculosis.^29^

### Culture

Compared to microscopy, culture has a higher sensitivity and it is capable of differentiating tuberculous from nontuberculous mycobacteria, although it takes relatively longer to have test results and is more expensive to carry out.^25^

The conventional use of culture has been performed on solid media until the invention of liquid media systems which used radioactive growth indicators and later the fast, efficient and sensitive fluorometric assays. Liquid media is relatively more sensitive and yields results faster in a matter of days compared to solid media which takes about 4 to 6 weeks.^30^ It can be used to perform drug susceptibility testing (DST), making it the preferred method, especially in high-income countries.^31^ One disadvantage of liquid culture is that it can be easily contaminated, hence affecting results. Culture-based methods remain the gold standard for diagnosing TB and for DST, even in the advent of rapid molecular tests.^32^ It is used in addition to sputum smear microscopy in making final clinical judgement.^23^

### GeneXpert

The increasing incidence and case-fatality rates of both MDR-TB and TB/HIV necessitated the need for diagnostic methods that are quick, easy to perform and affordable, especially for resource-constrained areas. In 2004, the GeneXpert system was established, and it brought about novel ways of performing polymerase chain reaction (PCR) based tests by simplifying and automating all the processes in performing the test. The Xpert^®^ MTB/RIF assay was finally completed in 2009 and made it possible to perform molecular tests without the need for standard laboratory settings. The Xpert^®^ MTB/RIF assay is a nucleic acid amplification test (NAAT) that can be performed directly on sputum and a few extrapulmonary specimens such as cerebrospinal fluid.^33^

The first time it was recommended by the WHO was in 2010 and subsequently in a policy update published in 2013. The updated guidelines gave recommendations on the use of Xpert^®^ MTB/RIF to diagnose TB in children, pulmonary TB (PTB), extrapulmonary TB (EPTB), multi-drug resistant TB (MDR-TB) and TB among persons with HIV.^31^,^34^, The technology can be used to diagnose TB and identify rifampicin-resistance (RR) in no more than two hours. It has demonstrated to have a higher sensitivity for smear-negative culture-positive TB, hence, useful in detecting TB among persons with HIV. The technology can be used at low levels of the laboratory network since its biosafety requirements are comparable to that of smear microscopy.^33^

Useful as it is, however, it does not replace the need for microscopy, culture and DST in monitoring treatment and detecting resistance to anti-TB agents apart from rifampicin.^31^ More recently, the WHO has issued a recommendation that the GeneXpert Ultra can be used in place of GeneXpert MTB/RIF in all settings.

The Xpert Ultra showed better performance in detecting TB in difficult-to-diagnose populations like persons living with HIV, children and in extrapulmonary TB. The sensitivity was up to 17% higher.^35^ There has been a 45% surge in case detection in patients infected with HIV with the introduction of the Xpert MTB/RIF test, which has a greater sensitivity than sputum smear microscopy thus resulting in significant strides made not only in TB diagnosis but also in the detection of isoniazid and rifampicin resistance cases.^36^,^37^

The introduction of GeneXpert as a TB diagnostic test has led to many countries modifying their TB policy implementation strategies. South Africa, Swaziland, Brazil and Moldova use GeneXpert MTB/RIF as the initial diagnostic test for all suspected cases whilst countries like the Philippines uses the GeneXpert MTB/RIF test following an abnormal digital chest X-ray and smear-negative sputum on microscopy.^38^,^39^

It has been documented that the GeneXpert MTB/RIF assay test increases in TB cases detected given its higher sensitivity compared to microscopy; thus, it increases the numbers of bacteriologically positive cases. There is also an increased detection of Rifampicin-resistant TB cases.^39^. Due to its short turnaround time, Gene Xpert MTB/RIF tests reduce diagnostic delay and time to treatment initiation, thus reducing morbidity and mortality associated with TB disease. Overall the test is said to be cost-effective especially in high burden settings.^38^,^39^

### Line Probe Assay

The line probe assay (LPA) (which also uses molecular tests) was recommended for the first time in 2008 by the WHO. They can detect resistance to rifampicin and isoniazid, with results being ready in up to 48 hours.^40^ The second-line line probe assay (SL-LPA) was introduced to test for resistance to second-line injectable drugs and fluoroquinolones after RR-TB or MDR-TB have been diagnosed. This development was expedient since the prescription of the shorter standardised MDR-TB regimen requires the exclusion of resistance to second-line regimen before initiation, as recommended in the WHO guidelines for drug-resistant TB (DR-TB).

After having these tests done, the clinician can determine the appropriate treatment regimen for patients to optimise outcomes without having to resort to trial and error. The SL-LPA is recommended in place of phenotypic DST as the initial test after establishing MDR-TB or RR-TB. One limitation of the test that requires more scientific enquiry is that though the test can identify mutations in the genes, they may not mean that the MTBC strain is resistance to all drugs that fall under a particular group.

We are yet to fully understand the different levels of resistance or cross-resistance that exist.^41^ Another limitation is they can only be performed in laboratories that rank high in the network, like central or national reference laboratories.^42^

### Lipoarabinomannan assay (LAM)

TB diagnosis among HIV positive patients can be challenging. Sputum microscopy and the classical radiological signs are often not the case. Also, HIV-TB coinfected may present with extrapulmonary symptoms. 43 The lateral flow lipoarabinomannan (LF-LAM) assay with a higher sensitivity even in severely compromised HIV patients. It detects the presence of lipoarabinomannan (LAM) antigen in urine which is present in people with active TB.^44^,^45^ The test is relatively easy to perform, cheap and needs little biosafety protocols as the risk of infection from the collection of sample is low compared to sputum smear microscopy.

The application of LF-LAM could improve early detection of TB in people living with HIV and prevent delays in the initiation of anti-TB treatment. The WHO recommends the use of LF-LAM to help diagnose TB only among persons with HIV infection with CD4 count less than 100 cells/mm^3^ who are seriously ill or persons with HIV who are seriously ill irrespective of CD4 cell count. The recommendation also suggests using the test in children, although there are concerns about its specificity among children. The use of other diagnostic tests like Xpert MTB/RIF, culture or sputum smear microscopy is encouraged since they exceed the LF-LAM in diagnostic accuracy in general.^46^

### Tuberculosis Screening and Diagnosis Protocol in Ghana

Until recently, presumptive pulmonary TB diagnosis was mainly by sputum smear microscopy in Ghana. This is readily available and has widespread use in most government health facilities in the country. TB is considered as a disease of public health interest; the cost of diagnosis and treatment is free to all patients. Currently, the GeneXpert MTB/RIF assay test is the recommended first-line in Ghana.

With the introduction of GeneXpert MTB/RIF assay test, screening for any presumptive TB cases is first by a digital X-ray and later with GeneXpert MTB/RIF if indicated. This has led to fewer laboratory request for TB cases as compared to the era where Smear Microscopy was used as the screening and diagnostic tool in Ghana.

## Discussion

### Clinical Management

#### TB preventive therapy

The WHO defines latent TB infection as: *“a state of persistent immune response to stimulation by Mycobacterium tuberculosis without evidence of clinically manifested active TB”.* It is estimated that about one-third of the world's population has latent TB infection. The progression of latent infection to active disease is influenced by factors such as immunosuppression (which may sometimes be caused by HIV infection) and malnutrition.

Latent infection, when detected, can be treated with isoniazid to prevent the development of active TB disease. Isoniazid preventive therapy (IPT) is recommended for HIV-positive persons and children under five years who have household contact with bacteriologically confirmed TB patients. Though isoniazid preventive therapy is strongly recommended as part of the basic HIV care package, it is poorly implemented in several countries.^47^ Poor implementation can be attributed to issues of the supply chain, challenges excluding active TB, financing of drug costs, unfounded concerns about isoniazid among others.^48^,^49^

Implementation of isoniazid prophylaxis as part of the HIV-TB response is extremely low. Actual coverage of IPT among HIV infected persons in Ghana is lacking.^12^ It is expected that HIV infected infants who are less than one year with or without household contact of active TB patients receive isoniazid for six months as long as they are declared to be without active TB disease. Monotherapy of isoniazid lasting six months is recommended for the treatment of LTBI in adults and children. Other alternatives include daily isoniazid and rifampicin for three months for children and adolescents less than fifteen years. Rifapentine and isoniazid may also be given weekly for three months for adults and adolescents.^50^

### Cotrimoxazole preventive therapy

The WHO provisionally recommended the use of fixeddose combination of trimethoprim-sulphamethoxazole (cotrimoxazole) for preventive therapy in people living with HIV in Africa in the year 2000. The prophylaxis was to be given to asymptomatic adults with CD4 counts less than 500 cells/mm^3^, symptomatic individuals at stage two of HIV disease or higher and pregnant women in second or third trimesters. Cotrimoxazole was also recommended for infants six weeks or older exposed or proven to be infected with the virus.^51^ These recommendations came a year after a randomised trial conducted in Cote D'Ivoire reported a decline in mortality of 48% among people living with HIV who received cotrimoxazole prophylaxis compared to those who received placebo.^52^

Later in 2006, guidelines for national programmes of resource-limited settings were developed. For people living in malaria endemic regions, a threshold CD4 count of < 350 cells/mm^3^ was indicative of cotrimoxazole preventive therapy for adults and adolescents to prevent malaria and other bacterial infections. It was also recommended for persons with WHO stages three or four of HIV infection and also persons with extrapulmonary TB.^53^ Currently, WHO recommends that cotrimoxazole preventive therapy be given to all TB/HIV patients as soon as possible and throughout anti-TB treatment. The prophylaxis is given to prevent the breakout of opportunistic infections that are common among persons living with HIV and is encouraged among people with TB/HIV co-infection in combination with ART where it gives continual protection against bacterial infections, diarrhoea and malaria in Africa.^54^

### Timing of ART initiation in co-management of TB/HIV

CD4 cell count played an important role in the care and management of opportunistic infections in HIV/AIDS. Until recently, CD4 cells was used in determining the initiation of ART. This was when drug toxicity, affordability and availability were major issues of concern in the late 1990s.^55^ In 2002, the CD4 threshold for starting ART was less than 250 cells/mm^3^.^56^ The drug combinations used then had high pill burden, less effective and associated with high risk of toxicity and resistance.

With time, more effective, less toxic, and affordable combinations were developed which paved way for research into the optimal CD4 threshold to initiate ART. More studies reported the advantages of initiating ART at higher CD4 thresholds and they began to reflect in the 2010 WHO guidelines, where the threshold was raised to 350 cells/mm^3^, then to 500 cells/mm^3^ in 2013.^55^ Recent studies show that starting ART among persons with CD4 above 500 cells/mm3 is beneficial and even reduces the risk of transmission to other persons.^57^ The latest WHO guidelines, therefore, recommend early initiation of ART, hence treating all patients, irrespective of CD4 count, and as soon as a confirmed diagnosis of HIV infection is made.^54^

Nevertheless, a baseline CD4 count is required to determine the stage of the disease. The use of viral load tests is preferred in monitoring disease progression and response to treatment.^55^ A clinical trial conducted in Cote D'Ivoire showed the benefit of co-treatment with ART and IPT on reducing TB events among persons with HIV. However, the change in CD4 counts did not differ significantly between patients who were given IPT and those who were not.

The study showed that early initiation of ART and six months of IPT given concomitantly was safe and that this therapy reduced the risk of tuberculosis events and other HIV-related comorbidities by 44 % and mortality by all causes by 35% compared to administration of ART alone.^58^ The third edition of the WHO treatment guidelines for TB, which was published in the year 2003 gave four possible options for the timing of ART initiation for TB/HIV co-infected patients.

The options included:

- initiating ART after the full course of anti-TB treatment was completed,
- initiating ART after the initial phase of anti-TB treatment was completed, and giving ethambutol and isoniazid in the continuation phase,
- using a rifampicin-based regimen in addition to two nucleoside reverse transcriptase inhibitors (NRTIs) or
- using a rifampicin-based regimen in addition to two NRTIs, then upon completion of anti-TB treatment, change ART regimen to a maximally suppressive HAART.^59^

Anti-TB treatment is prioritised over ART in order to stop transmission of TB (especially in the case of smear-positive PTB), however, there is a need to treat both TB and HIV concurrently in order to maximise survival benefit. Patients with disseminated TB or with CD4 counts less than 200/mm^3^ were eligible for concurrent initiation of both ART and anti-TB medications back in 2004, whilst those who showed no signs of serious illness had to defer ART until after the initial phase of anti-TB treatment.^60^

Early initiation of ART reduces mortality among TB/HIV co-infected patients, 60,61 informing later recommendations of the WHO on starting ART for all HIV positive patients regardless of CD4 cell count as soon as possible or within the first eight weeks of beginning anti-TB regimen. The timing of ART initiation in this instance is very crucial to the survival of people with dual infection since deaths have been shown to occur mostly within the first two months or during the intensive phase of anti-TB treatment. The latest guidelines, however, stress on starting ART within the first two weeks of anti-TB treatment for patients with CD4 counts less than 50 cells/mm^3^ and within eight weeks for persons with higher CD4 counts.^54^ The exact timing of ART for TB and HIV co-treatment still remains unclear, as clinicians deal with adverse reactions, adherence, immune reconstitution and other problems that are associated with it.

### TB-HIV in children

The diagnosis of PTB in children is difficult, more so in HIV associated PTB. The presence of other pulmonary diseases related to HIV infection, atypical chest x-ray findings and young age (1 to 5 years) are just few of several factors that make detection of TB difficult.^59^ The diagnosis and treatment of TB is same for all children irrespective of HIV status. The regimen is also same for adults, however, dose adjustment is made based on body weight.^62^

As in adults, the recommendation is to treat all children with HIV regardless of CD4 count. The timing of ART in HIV associated TB is like that recommended for adults. For children with CD4 less than 50 cells/mm^3^ ART must be started within two weeks of anti-TB. Cotrimoxazole preventive therapy is also recommended as part of integrative therapy of TB/HIV management in children.^63^

### Management of PTB and EPTB (TB meningitis)

Anti-TB regimen given to HIV-positive patients is same for HIV-negative patients. Thioacetazone used in the 1990s was replaced with ethambutol because of severe adverse reactions on the skin, which was sometimes fatal in HIV patients.^27^,^59^,^63^

Treatment of TB is in two phases: the initial/intensive phase where a combination of four medications are given, lasting for two or three months for first and second-line treatment, respectively. The goal of the intensive phase is to greatly reduce the bacterial burden and render the patient non-infectious. The continuation phase (combinations of two drugs are used), which also lasts for four to six months, depending on the regimen given aims at sterilising or clearing the patient of the mycobacteria. This is known as the short-course therapy.^27^

The main drugs used in the treatment of TB include isoniazid (H), rifampicin (R), pyrazinamide (Z), and ethambutol (E) and streptomycin (S) (no longer a key part of short-course therapy). The earlier versions of the treatment guidelines recommended an intensive phase of two months' daily dose or an intermittent dose of HRZE/HRZS and six months of HE or four months of HR depending on the formulation chosen by the respective national program and resources available in the country. It was thought at that time that intermittent intake (three times weekly) was as efficacious as daily intake.^59^,^63^

A systematic review by Khan et al., showed that intermittent intake was associated with higher rates of treatment failure or relapse compared to daily dosing.^64^ The WHO in their 2010 publication of treatment guidelines therefore recommended daily intake of drugs.^27^

Furthermore, new patients who test positive for sputum smear examination at the end of the intensive phase can now move to the continuation phase without extending the intensive phase (as was the standard previously) as long as treatment regimen contains rifampicin.^3^,^27^

HIV infected patients often present with extrapulmonary TB signifying an advanced disease. HIV-associated extrapulmonary TB is classified as WHO clinical stage four of HIV infection (AIDS). It may present as disseminated TB, TB associated pericarditis, lymphadenitis, meningitis, among others. The diagnosis of extrapulmonary TB can be challenging and often require experienced physicians to make accurate diagnosis as suggested in the Ghanaian TB management and care guidelines.^23^

The management of extrapulmonary TB is essentially the same as that of pulmonary TB, although the duration of treatment may be prolonged for up to nine to twelve months in the former.^27^ The use of adjuvant corticosteroids such as dexamethasone or prednisolone for six to eight weeks for persons with TB meningitis and TB pericarditis is recommended, although questions on the optimal dosage, duration and its different effects on HIVpositive (on ART and not on ART) as well as HIV-negative people are yet to be answered.^54^

### MDR TB and HIV management

The first- ever national drug resistance survey in Ghana in 2016; reported the proportions of drug-resistant TB to be 1.5% and 7% for new cases and previously treated cases, respectively.^13^ The resistance of certain MTBC organisms to isoniazid and rifampicin characterises multidrug resistance TB; resistance to these two drugs extending to any fluoroquinolone and at least one of the second line injectable drugs is extensively drug-resistant TB. These two conditions greatly threaten the progress needed to realise the goals set in the End TB Strategy. Coupled with HIV infection, there is an increased risk of adverse treatment outcome with case-fatality rates, sometimes exceeding 90%, especially when the regimen is without ART. It is therefore important to screen all TB/HIV patients for resistance before starting TB regimen in order to optimise outcome by providing the appropriate treatment regimen. On the contrary, universal screening may not be feasible in resource-limited settings, in which case DST may be limited to patients with treatment failure or those who are at a high risk of drugresistant TB.^54^

A conventional regimen of at least 20 months of therapy was initially recommended for all MDR-TB cases 40,65 until recently when there was a movement towards a shorter duration of nine to twelve months for patients who have not been treated previously with the second-line anti-TB regimen and who have no resistance to fluoroquinolones and second-line injectable drugs.^66^

With regards to the timing of ART during treatment of MDR-TB, some questions remain unanswered. Early guidelines suggested initiating ART at two weeks of anti-TB treatment for individuals with CD4 less than 200 cells/mm^3^ and eight weeks for those with CD4 between 200 and 350 cells/mm^3^.

WHO treatment guidelines for drug-resistant tuberculosis recommend ART for all HIV positive MDR-TB cases irrespective of CD4 count between two and eight weeks (as early as possible) of starting anti-TB regimen, though the quality of evidence for this recommendation is low.^65^,^66^ The optimal timing of ART for HIV associated MDR-TB is still unknown. The concerns are quite different in patients who are diagnosed with drug-resistant TB (DR-TB) while on ART. If ART failure is detected to be the cause of TB disease, the ART regimen is changed to second-line between two to eight weeks after beginning DR-TB treatment.^67^

### Immune reconstitution inflammatory syndrome (IRIS)

The commencement of ART in TB/HIV co-infected patients while on anti-TB regimen can sometimes result in a paradoxical reaction due to rapid recovery of immune function that leads to an inflammatory reaction. This reaction usually occurs within three months of ART regimen. It is often among patients who are initiated on ART at an early stage. Characteristic features include exacerbation of active TB symptoms like cough, night sweats, tuberculous abscesses, and enlargement of lymph nodes. The other form of IRIS (unmasking), occurs in patients with subclinical TB that becomes reactivated after initiating ART.^68^,^69^

Patients with advanced HIV infection may also show similar signs and symptoms such as treatment failure or drug resistance, making the diagnosis of IRIS challenging. The occurrence of paradoxical reactions is not exclusive to persons with dual infection. Ten per cent and 28% of HIV-negative and HIV-positive patients developed paradoxical reactions in one study.^70^ The diagnosis of IRIS can be made after excluding other conditions which may be responsible for clinical deterioration. Its management usually depends on the degree of severity of the signs and symptoms. ART may be continued whilst treating IRIS with one of several options, including corticosteroids or non-steroidal anti-inflammatory drugs.^67^ Continuation of anti-TB and ART is encouraged and close monitoring critical in optimising outcome.

## Conclusion

The syndemic interaction between Tuberculosis and HIV has contributed significantly to morbidity and mortality globally; disproportionately affecting Africans. Understanding the dual biology of HIV and TB co-infection together and evidence-based approaches for clinical management is essential for improved outcomes. Although adequate control of the TB-HIV co-infection is a daunting task, identification of cases of co-infected patients, including MDR-TB, is an important step to providing optimal treatment and elimination of TB and HIV.

## Figures and Tables

**Figure 1:**
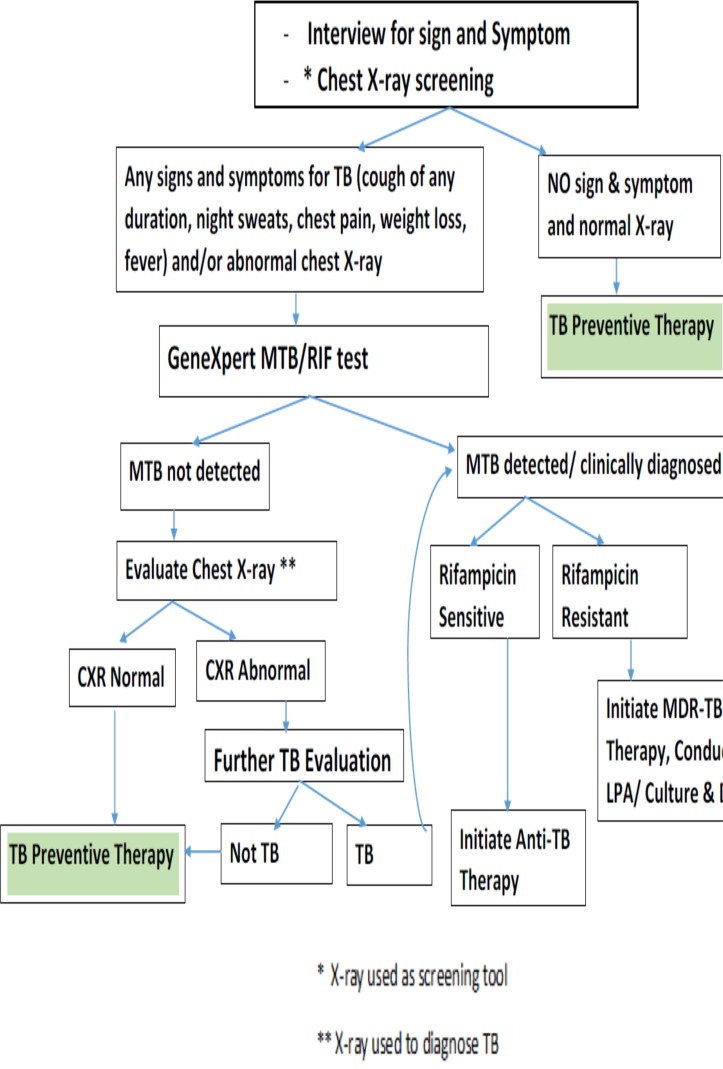
Algorithm for screening and diagnosis TB in people living with HIV in Ghana (Source NTP, 2018).

**Figure 2:**
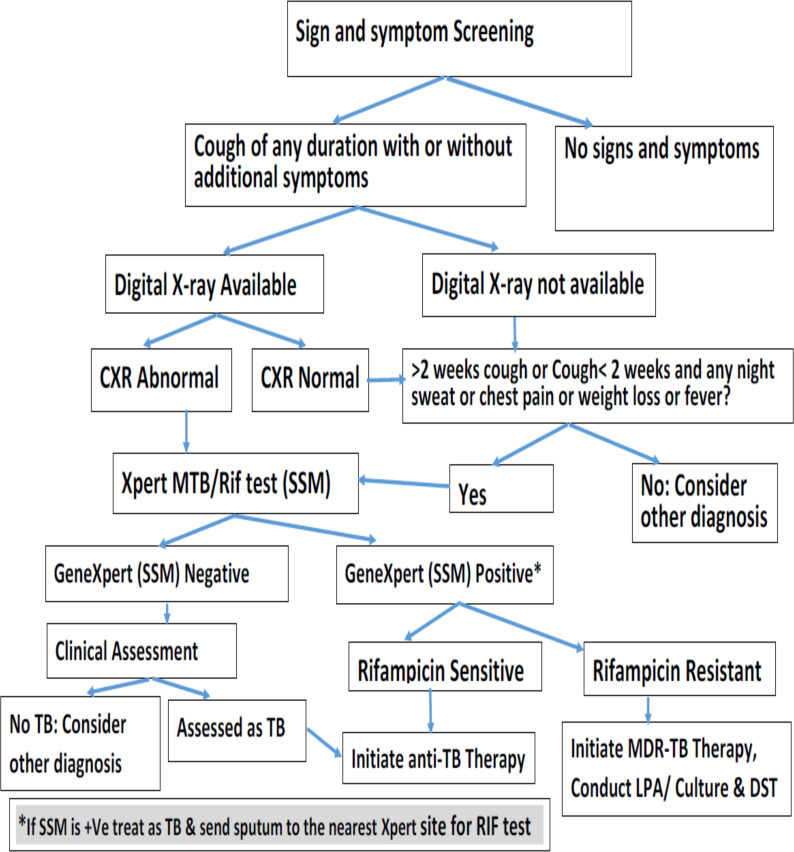
Algorithm for screening and diagnosis TB for facility-based case finding in Ghana (Source NTP, 2018).

**Figure 3:**
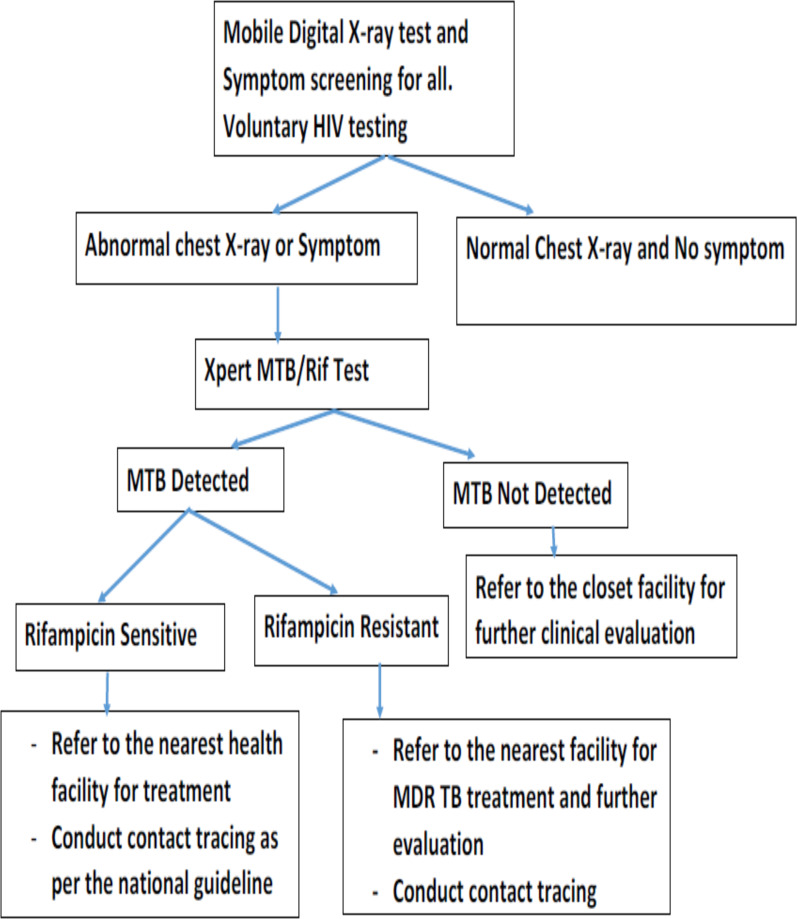
Algorithm for screening and diagnosis TB for Community Targeted screening in Ghana (Source NTP, 2018)
